# Lesser-known or hidden reservoirs of infection and implications for adequate prevention strategies: Where to look and what to look for

**DOI:** 10.3205/dgkh000247

**Published:** 2015-02-04

**Authors:** Sally Bloomfield, Martin Exner, Hans-Curt Flemming, Peter Goroncy-Bermes, Philippe Hartemann, Peter Heeg, Carola Ilschner, Irene Krämer, Wolfgang Merkens, Peter Oltmanns, Manfred Rotter, William A. Rutala, Hans-Günther Sonntag, Matthias Trautmann

**Affiliations:** 1 London School of Hygiene and Tropical Medicine, International Scientific Forum on Home Hygiene, London, UK; 2 Institute of Hygiene and Public Health, Bonn University, Bonn, Germany; 3 Department of Chemistry, University of Duisburg-Essen, Essen, Germany; 4 Schülke & Mayr GmbH, Norderstedt, Germany; 5 Departement Environment et Santé Publique S.E.R.E.S., Faculté de Médicine, Nancy, France; 6 Institute of Medical Microbiology and Hygiene, Tübingen, Germany; 7 Pharmacy Department of Mainz University, Mainz, Germany; 8 Hygiene Institute of Vienna University, Vienna, Austria; 9 UNC School of Medicine, Chapel Hill, North Carolina, USA; 10 Institute of Hygiene and Medical Microbiology, Heidelberg, Germany; 11 Department of Hospital Hygiene at Stuttgart Hospital, Stuttgart, Germany

**Keywords:** Infection reservoir, pathogen detection method, outbreak, hygiene, viable but nonculturable status, prevention strategy

## Abstract

In developing hygiene strategies, in recent years, the major focus has been on the hands as the key route of infection transmission. However, there is a multitude of lesser-known and underestimated reservoirs for microorganisms which are the triggering sources and vehicles for outbreaks or sporadic cases of infection. Among those are water reservoirs such as sink drains, fixtures, decorative water fountains and waste-water treatment plants, frequently touched textile surfaces such as private curtains in hospitals and laundry, but also transvaginal ultrasound probes, parenteral drug products, and disinfectant wipe dispensers.

The review of outbreak reports also reveals Gram-negative and multiple-drug resistant microorganisms to have become an increasingly frequent and severe threat in medical settings. In some instances, the causative organisms are particularly difficult to identify because they are concealed in biofilms or in a state referred to as viable but nonculturable, which eludes conventional culture media-based detection methods. There is an enormous preventative potential in these insights, which has not been fully tapped. New and emerging pathogens, novel pathogen detection methods, and hidden reservoirs of infection should hence be given special consideration when designing the layout of buildings and medical devices, but also when defining the core competencies for medical staff, establishing programmes for patient empowerment and education of the general public, and when implementing protocols for the prevention and control of infections in medical, community and domestic settings.

## Zusammenfassung

Bei der Konzeption von Hygienestrategien standen in den letzten Jahren die Hände als Hauptübertragungsweg für Infektionen im Mittelpunkt. Es gibt jedoch noch eine Vielzahl weiterer, weniger bekannter und unterschätzter Infektionsquellen für Mikroorganismen, die als Verursacher und Infektionsweg für Ausbrüche oder sporadische Infektionsgeschehen in Frage kommen. Zu diesen zählen wasserführende Systeme wie Waschbeckenablauf, Armaturen, Zierbrunnen und Kläranlagen, textile Kontaktflächen wie Patientenvorhänge in Krankenhäusern und Wäsche, aber auch transvaginale Ultraschallsonden, parenteral verabreichte Medikamente und vorgetränkte Tuchspendersysteme für Flächendesinfektionsmittel.

Eine Analyse vorhandener Berichte über Ausbrüche zeigt zudem, dass Gram-negative und multiresistente Erreger eine zunehmend häufige und ernste Gesundheitsgefahr im medizinischen Umfeld darstellen. In einigen Fällen ist es besonders schwierig, den Infektionserreger zu finden, weil er sich in Biofilmen verbirgt oder im sogenannten VBNC-Status befindet – viable but nonculturable (VBNC) bedeutet lebensfähig, aber nicht kultivierbar. In diesem Status entzieht sich der Mikroorganismus den konventionellen, auf Kultivierbarkeit basierten Nachweismethoden. In diesen Erkenntnissen liegt ein großes Präventionspotential, das noch nicht ausreichend genutzt wird. Neue und wiederauftretende Krankheitserreger, neuartige Nachweismethoden und verborgene Infektionsquellen sollten daher zukünftig größere Beachtung finden, auch wenn es darum geht, neue Medizinprodukte zu entwerfen oder Gebäude zu planen. Diese Erkenntnisse müssen mit einfließen in die Kernkompetenzen medizinischen Personals, in die Patientenaufklärung und die Aufklärung der Allgemeinbevölkerung sowie in die Strategien für die Prävention und Kontrolle von Infektionen in medizinischen und öffentlichen Bereichen sowie im privaten Umfeld.

## Background

Recent reports of severe outbreaks with pathogens from previously unrecognized or underestimated reservoirs and with antibiotic-resistant organisms have become a growing concern for the general public as well as for the scientific community. Following an invitation by Rudolf Schülke Foundation, a panel of 13 internationally renowned experts met for a two-day symposium to explore lesser-known infection reservoirs and crucial elements for the design of suitable infection prevention and control strategies. A multidisciplinary approach was taken comprising scientific views from specialists in chemistry, microbiology, pharmacy as well as hospital hygiene. The attendees identified three key points for a more in-depth discussion: acknowledging latent pathogens, identifying and raising awareness of underestimated and lesser-known infection reservoirs, and implications for prevention strategies. The most important findings, corroborated by examples from recent scientific literature, are summarized in the report below.

## Latent pathogens: characteristics and implications of microorganisms in the VBNC state

### Viable but nonculturable

One of the major issues in risk assessment preceding infection prevention and control is the quantitative determination of pathogens. Vice versa, the efficacy of infection prevention and control strategies such as disinfection and sterilization is largely based on measuring the quantitative reduction in the microbial load. Current routine test systems for detecting microorganisms such as agar plating, membrane filtration and broth enrichment are growth-dependent. These tests presuppose certain metabolic activities and are designed to find “viable” microorganisms, which can multiply on culture media. Viability in this context is usually regarded to equal “being alive”. Hence, microorganisms are commonly considered non-viable and dead if they do not multiply on culture media.

In the early 1980s, however, a special property of bacteria was identified which is referred to as the “viable but nonculturable (VBNC) state” [1]. According to the current definition, “a bacterial cell in the VBNC state is one which fails to grow on the routine bacteriological media on which it would normally grow and develop into a colony, but which in fact is alive and metabolically active” [2]. These VBNC populations have the potential to act as hidden reservoirs of infection. For example, Powers et al. investigated contact lens decontamination products and protocols and found that a medically significant amount of bacteria remained on the contact lenses after disinfection and/or cleaning, which were viable but nonculturable and remained undetected when using standard culture methods [3].

The VBNC state is usually entered in response to environmental stress factors such as extreme temperatures, presence of heavy metals, low oxygen content, changes in pH, or presence of (food) preservatives. On the other hand, seemingly “dead” bacteria may simply be latent or dormant in their VBNC state and may be resuscitated when exposed to favourable conditions such as temperature upshift [4]. Also, higher organisms such as Acanthamoeba may act as mediators for resuscitation from the VBNC state, e.g. for *L. pneumophila* [5]. 

Most importantly, microorganisms in the VBNC state can resume growth and also regain their infectivity. 

Species which have been described to enter this state are Gram-positive species (e.g. *Enterococcus, Listeria*) as well as Gram-negative species (e.g. *E. coli, Legionella pneumophila, C. jejuni, S. enterica, P. aeruginosa, H. pylori*) [4]. More recently, yeasts, in particular *S. cerevisiae*, have been discovered to be capable of entering and also exiting the VBNC state (e.g., [6]). 

### Characteristics of the VBNC state

Life signs of cells include maintenance of the intracellular environment, presence of intact nucleic acids, membrane potential, efflux pump activity, enzyme presence, substrate uptake/incorporation, and others [7]. Physiological changes in VBNC cells may, for example, occur in the composition of the outer membrane structure [8], cell protein profiles [9], and membrane fatty acids. Metabolic activity is maintained, although at a low level [10], [11]. Typical VBNC-associated morphological changes of microorganisms include reduction in size (“dwarfing”) and/or formation of o-shaped or coccoid structures, but enlargement has been observed, too (e.g., for *Campylobacter jejuni* [12]). 

Changes in properties may also occur. A recent study demonstrated that *V. vulnificus* in the VBNC state has a higher resistance to a variety of challenges, including heat, low pH, ethanol, antibiotic, heavy metal, oxidative and osmotic stress, than those growing in exponential phase [13].

Generally, entering the VBNC status is viewed as a survival strategy of infectious as well as intoxicating microorganisms.

Current detection methods for microorganisms in the VBNC state include direct viable count (DVC), polymerase chain reaction (PCR), ATP bioluminescence, flow cytometry and fluorescence in situ hybridization (FISH) with fluorescent labelled gene probes. VBNC microorganisms exist in aquatic environments, in biofilms, in foodstuff, on surfaces and in the human body and thus might have an impact on a variety of public health areas including the manufacture of medicinal products and the food industry. 

### Evidence of resuscitation and regained infectivity

One of the VBNC microorganisms which has been studied in greater detail is *P. aeruginosa*. The strains can be found in biofilms where they are made visible by applying the FISH method. Dwidjosiswojo et al. [14] observed that *P. aeruginosa* strains enter the VBNC state in response to stress induced by copper ions present in plumbing systems for drinking water. While the total number of bacterial cells in the system remained unchanged and the membrane remained intact (“life sign”), culturability of *P. aeruginosa* drastically decreased. Loss of culturability depended on copper (Cu) concentration and exposure time. When DDTC (diethyldithiocarbamate) was added as chelating agent to the copper-stressed bacteria in order to inactivate Cu, all cells resuscitated after 14 days and the cytotoxic effect of the revived cells was confirmed [14]. 

The virulence of cells during the VBNC phase varies depending on the microbial species and a number of other (environmental) factors (cf. [13], [15]).

### Implications

Although there is increasing awareness that not being culturable is not a proof of being dead for a microorganism, the questions remain as to how microbial death should be defined, and what the implications of microorganisms in the VBNC state are in terms of public health risks and infection control methods. Many other questions have been raised which still need final answers, e.g., regarding the prerequisites for resuscitation, the infectivity of cells during the VBNC state, the effects in relation to disinfectants and to antibiotics exposure, and how the VBNC state may affect routine methods of detection, and so on. In efficacy testing of disinfectants, it is known that the choice and concentration of the neutralising medium can have a profound impact on the numbers of microorganisms which survive and replicate. While there is a potential risk of underestimating the microbial bioburden on contaminated surfaces and overestimating the effects of disinfection and sterilization procedures when applying standard microbiological methods, these routine methods should not be abandoned unless or until validated methods of distinguishing viable (or infectious) from non-viable organisms become available. Current methods are based on long-term experience and have proven to be a valuable tool for establishing and monitoring effective hygiene precautions and thus still constitute the mainstay for pathogen detection and enumeration.

## Underestimated and lesser-known reservoirs of infection

Management of outbreaks is often compromised by the persistence of the causative organisms. One reason may be that pathogens are difficult to detect with conventional methods, e.g., because they entered the VBNC state and are concealed in biofilms. Sometimes, however, the actual reservoir of infection is not identified because the infection risk of a particular source is either unknown or not taken into consideration.

### Water as a reservoir of infection

#### Assessment of infection risks associated with waterborne pathogens 

A multitude of bacteria, viruses, protozoa and helminths have been reported to live in aqueous habitats with the potential to cause infectious diseases such as diarrhoea, cholera, typhoid and others. Among the better known infectious agents are Hepatitis A Virus, Norovirus, *Legionella* spp., *E. coli, P. aeruginosa, Vibrio cholera, Schistosoma* spp., and moulds (cf. World Health Organization Guidelines for drinking-water quality, Table 7.1 [16]). Less is known about opportunistic pathogens such as Nocardia and Mycobacterium (non-tuberculosis species). The routes of transmission are primarily by ingestion (drinking), but also by inhalation and aspiration (aerosols) and by direct contact (bathing). Insects breeding in water also contribute to the outbreak of diseases, serving as vectors, e.g., for dengue fever and malaria. The persistence of pathogens varies with species, environmental conditions such as biofilm formation or possibly survival within amoeba, and favourable living conditions such as stagnant water and growth-supportive temperatures or pH values.

Infection risk assessment should include a dose-response evaluation, which investigates the quantitative relationship between the (ingested, inhaled) dose of a pathogen and the probability for disease manifestation. Based on the results, “tolerable infection risks” and the acceptable levels of specific microorganisms in the drinking water can be defined [17]. However, reliable dose-response calculations do not exist for all transmission routes and microorganisms. Dose-response calculations for *Legionella pneumophila* in aerosols have recently been made [18].

Other points to consider in risk assessment are frequency and duration of exposure, transmission pathway, (tap) water source, distribution/plumbing system (including warm water reservoirs), and the immunological status of the patient. For example, Mena and Gerba identified two routes of infection which appear to carry the greatest health risks from contacting water contaminated with *P. aeruginosa*: skin exposure in hot tubs and lung exposure from inhaling aerosols [19]. The risk of infection from ingesting *P. aeruginosa* contained in drinking water is low for healthy individuals, although the oral median infective dose increases with antibiotic treatment. The dose-response relationship to dermal exposure to *P. aeruginosa* contained in drinking water has not been defined.

#### Examples for waterborne Legionella infections in non-medical settings

The European Centre for Disease Prevention and Control (ECDC) and the U.S. Centers for Disease Control and Prevention (CDC) report increasing incidences of Legionnaires’ disease, especially community-acquired cases [20], [21]. Transmission via aerosols, often extending over long distances (up to 6 km and more), is the most common pathway and must also be taken into consideration when searching for the infection reservoir. Other well-known waterborne pathogens in community settings include norovirus and *Cl. difficile* [22], [23]. 

Frequently, legionellosis outbreaks have been associated with cooling towers or hot tubs, but also with water fountains, potting soil and humidifiers [24], [25], [26], [27], [28]. It may take a long time, before the source of infection is found: Biofilm formation in a sulphur-rich warm spring on site was only recently traced to be the potential source of successive outbreaks of legionellosis in the same French thermal spa in 1986, 1994, and 1997 [29]. An example for a previously unknown infection reservoir for legionella is a wastewater treatment plant, which was found to be associated with the contamination of cooling towers, resulting in a community-wide outbreak of legionellosis in Germany in 2013 in which 159 people were affected and two patients died [30]. 

It is important to realize that the reservoir of infection with legionellae is not always found. Outbreak reports are often incomplete and/or published only in local newspapers or as online news. Accounts of sporadic occurrences of legionellosis and other cases of waterborne infections in private homes are often overlooked (e.g., describing hot water tanks as infection reservoir [31], [32]). 

Decontamination practices may prove difficult as *L. pneumophila* can persist in biofilms and colonize within multispecies microbial communities. Much remains unclear as to whether their resistance to sanitizing strategies is due to the biofilm structure itself, their association with amoeba, or both [33] – or, possibly, their entry into the VBNC state.

Although there is a large body of data on outbreaks of waterborne diseases in non-medical settings, a systematic analysis of these reports and subsequent implementation of important insights in preventative measures is often lacking. In this context, the Mayor of Quebec, Régis Labeaume, may be quoted with his statement referring to the 2012 Legionella outbreak [28]: “This summer’s Quebec outbreak was all the more tragic because a report 15 years ago suggested ways to prevent it but was ignored.”

#### Waterborne-infections in the medical setting

As a result of the greater susceptibility of patients and residents of hospitals and/or long-term care or rehabilitation centers to infections, waterborne pathogens are more likely to cause infection in healthcare institutions than in the healthy population. Opportunistic pathogens such as *P. aeruginosa* or *S. maltophilia* can constitute a serious health hazard in these settings. A recent systematic review on the role of water in healthcare-associated infections [34] showed that plumbing systems, including sink drains and their fixtures, room humidifiers and decorative fountains have been implicated in severe outbreaks. 

However, many more reservoirs are possible but remain unknown because – again – they are not described in outbreaks or case reports. Reservoir detection requires meticulous investigation and long-time experience. The common reservoir of an infection outbreak can be difficult to pinpoint. It can be obscured by cross-infection, because colonized patients become a secondary source of infection, and because pathogen transfer takes a variety of routes. For example, in a protracted outbreak of multidrug-resistant* A. baumannii* infections, transmission from carriers most likely occurred via the hands of healthcare workers, poor cleaning protocols, airborne spread, and contaminated water from sink traps [35]. Similarly, in their review of the association between healthcare water systems and *P. aeruginosa* infections, Loveday et al. [36] concluded that although water systems are known to act as a source of infection, the exact route of transmission remains unclear. Contamination seems to be confined to the distal ends of a plumbing system. Even electronic faucets may become a reservoir [37] when biofilm formation is enhanced due to the use of plastic materials, reduced water flow, and a longer distance between valves and taps. 

Decorative water fountains are an example where infections can be easily prevented once the primary reservoir is identified. Several reports have shown infections to be related to water cascades and decorative fountains, even where standard maintenance and sanitizing methods were provided (cf. Table 1 (Tab. 1), [38], [39]). As a consequence, these fountains are now considered to present an unacceptable risk in hospitals serving immunocompromised patients.

### Endoscopes and ultrasound probes as infection reservoirs

Within the broad range of instruments which are used in healthcare settings, the processing of endoscopes continues to present a particularly serious risk of infection. In a 2013 review on infection rates following flexible endoscopy and bronchoscopy, based on an evaluation of nearly 100 publications, Kovaleva et al. [40] found upper gastrointestinal endoscopy to be associated with the highest risk. Reasons for reprocessing failures are manifold and include errors in manual processing, unrecognized endoscope wear and tear, use of a contaminated water supply during disinfection and final rinsing, or inadequate storage. Also, the importance of the cleaning step in processing is underestimated. Efficacy testing of the cleaning effect under use conditions is often unreliable, partly because the definition of cleanliness (“how clean is clean?”) is still subject to controversy. Measures for prevention of medical-device-associated infections should include adequate statutory regulations, proper training of the staff, validation of automated cleaning and disinfection procedures, standard operating protocols for manual processing steps, safe storage, monitoring of processing practices, and surveillance. Apart from these precautions, new designs of endoscopes with detachable, single-use channels may enhance the safety of endoscopic procedures [41].

Until recently, endocavity ultrasound has not been regarded a high-risk procedure with regard to infection transmission. However, following various current reports of contaminated transvaginal ultrasound probes, this has changed. Leroy [42] concluded from his systematic review and meta-analysis that there was a pooled prevalence of 12.9% (95% confidence interval: 1.7–24.3) for pathogenic bacteria, and 1.0% (0.0–10.0) for frequently occurring viruses (human papillomavirus, herpes simplex virus, and cytomegalovirus) for endovaginal/rectal probes, both after low-level disinfection (wipes and spray). A study by Casalegno et al. [43] revealed that a considerable number of ultrasound probes are contaminated with human and HR-HPV DNA, despite LL disinfection and probe cover. The authors therefore recommend high-level disinfectants such as glutaraldehyde or hydrogen peroxide solutions. For some settings, probe disinfection using closed automated systems (e.g., with hydrogen peroxide aerosol) may be a safe and feasible solution. However, to date no standardized disinfection protocols exist with respect to inactivating human papillomavirus under practice conditions. 

### Parenteral drug products as infection reservoirs

In the summer of 2010, three neonates in the perinatal intensive care unit of the Mainz University Hospital, Germany, died after i.v. administration of total parenteral nutrition (TPN) admixtures [44]. The TPN admixtures had been prepared on the day of administration in the cleanroom room environment of the hospital pharmacy department with meticulous adherence to aseptic procedures according to the Good Manufacturing Practice Guideline PIC/S PE10-03 for medicinal products in healthcare institutions. In-process controlling en compasses the daily preparation of dummy solutions/reference samples, which are usually stored for 14 days. Aliquots are transferred to blood culture bottles and sent for bacteriological testing. In this case, *Enterobacter cloacae* and *Escherichia hermanii* were detected in the samples the night following the preparation, and further administration of the admixtures was immediately stopped. The same strain of these bacteria was detected in all TPN admixtures as well as in the bulk infusion bottles of the amino acid solution, but not in the cleanroom area or pharmacy staff. By quantifying the viable bacterial count and the endotoxin concentration in the contaminated TPN mixture it was concluded that contamination must have occurred in the purchased glass bottled amino acid solution weeks or months earlier. The infection reservoir was eventually traced to a hairline crack in the glass bottle, which contained the amino acid solution. The crack most likely occurred during transport of the bottle in an unsuitable packaging system allowing bacteria to enter the solution from the outside. It was not until three years later that the company finally changed the packaging and transport system for the infusion solutions, even though these problems are easily preventable by placing a piece of cardboard between the bottles.

The elucidation of the source of infection in this outbreak demonstrates the value of thorough investigation and microbiological analysis, which examined all possibilities including those which might have seemed unlikely at the time, i.e. damaged primary containers of bulk solution. It also promoted the awareness to monitor transport problems as part of the quality assurance programmes during TPN compounding, since cracks or fractures in glass vials and other containers are not uncommon, but cannot always be discovered by visual inspection.

Strict adherence to the given guidelines for safe preparation of medicinal products should be emphasized. In a *Burkholderia cepacia* outbreak investigation among inpatients at Duke University Hospital in Durham, N.C., from August 31 through September 6, 2012, the anteroom sink drain and pH probe calibration fluid in the compounding cleanroom were detected as the reservoir of infection in the institutional pharmacy department [45]. Other large-scale outbreaks of infections linked to contaminated compounded parenteral medications (*S. marcescens* [46]) or glucocorticoid containing injection solutions (61 deaths associated with fungal meningitis) have also been described [47], [48].

### Disinfectant wipe dispensers as infection reservoirs

Bucket dispensing systems for pre-moistened surface disinfectant wipes have been identified as a potential infection reservoir, predominantly because of prolonged reuse periods of the disinfectant solutions. Contamination may occur e.g., if dosing units or potable water taps used to prepare the disinfection solution become contaminated with biofilms. Other critical control points are the use of wipes which are not compatible with the disinfectant or have been prepared with under-dosed disinfectant solutions. Dried out wipes or contaminated wipes hanging out of the bucket also represent risk factors [49]. When prepared and stored in inadequately processed receptacles for a long period of time, some disinfectant solutions (apparently especially those with surface-active ingredients) bear the risk of becoming a microbial reservoir, which predominantly involves gram-negative bacteria [50].

Although most available data are derived from microbiological studies, there is also a report of an outbreak report with *Klebsiella oxytoca*, where the pathogen was isolated from disinfectant buckets showing increased tolerance to the disinfectant solution (cf. [51], [52]). A recent investigation of an outbreak in a neonatal intensive care unit in Germany suggests the water line of a dosing device used for the preparation of pre-soaked surface disinfectant wipes to have served as one of the reservoirs for *K. pneumonia*, resulting in contaminated wipes (Exner, M., personal communication).

## Soft surfaces and laundry as infection reservoirs

### Privacy curtains

Among the infection reservoirs more recently described are soft surfaces such as furniture, mattresses, pillows and privacy curtains. Trillis et al. [53] found 42% of curtains surrounding patients’ beds to provide privacy to be contaminated with VRE, 22% with MRSA and 4% with *C. difficile* (also see [54]). Transmission of bacteria from curtains via the hands of healthcare personnel touching these curtains is possible ([55], [56] and chapter on laundry as infection reservoir below). In 2002, Das et al. reported an outbreak caused by a Carbapenem-resistant *Acinetobacter baumannii* in an intensive care unit of the tertiary-referral university teaching hospital in Birmingham with curtains surrounding patients’ beds as the major source [57]. A recent publication by Mahida et al. from Nottingham, U.K., describes an outbreak of invasive group A streptococcus infection (GAS) on an ear, nose and throat ward, where contaminated patient curtains were found to be the potential source of GAS cross-transmission, which had implications in relation to hand hygiene and frequency of laundering [58].

Possible decontamination practices include wiping the “grab area” of the curtain with improved hydrogen-peroxide containing disinfectants [59]. Increasing awareness of the problem is reflected in publications such as “Divider Curtains and Infection Risks” by the Canadian Comité sur les infections nosocomiales du Québec [60] at the end of 2013. A standard protocol for microbiologically safe use of hospital curtains has yet to be established.

### Laundry

Textiles as common-touch surfaces tend to get overlooked as infection reservoir because of the lack of intervention study data showing a direct link to infection. However, they also must be regarded as a potential vehicle of infection (before, during and after handling laundry), with the risk increasing where large quantities of pathogens are shed via vomit, faeces and skin, and where people have impaired immunity. This includes residential facilities for the elderly, where a hygiene regimen covering the whole process from collecting laundry to adequate storage is required. Apart from controlling infection risks, effective laundering is also important to prevent the spread of antibiotic-resistant skin and intestinal flora such as MRSA and multidrug resistant Gram-negative strains in domestic and medical settings. 

In 2011 IFH carried out a review of the data on infection risks associated with clothing, bedlinens etc. in community, hospitals and other healthcare settings [61]. The greater part of the data comes from studies showing how pathogens are shed onto, or transferred to, clothing etc., and the extent to which they can survive and spread to hands and surfaces such that we can become exposed to potentially infectious doses. The data show that viability on fabrics declines at a more or less rapid rate on dry clothing, depending on the microbial species and room humidity. Generally, survival of microbes on fabrics is significantly less than on non-porous contact surfaces. However, Gram-positive spp. such as *S. aureus, C. difficile* and fungal spp. can survive long periods (days to months) on fabrics. Survival times for Gram-negative species such as *E. coli *and* P. aeruginosa* are shorter, but survival times of up to 4 h or more have been recorded. Survival of viruses on fabrics is mostly around 30 min up to 12 h, up to a maximum of 48 h (no data are available for norovirus), whilst survival times for fungal species ranged from 1 day to several weeks. Transfer rates from moist fabrics to hands and other fabrics were around 1–10%, but in some cases, transfer was as little as 0.1% or less, or as high as 50% [55], [56]. Transfer rates varied according to microbial strain, temperature, room humidity, type of fabric and inoculum size. They are significantly lower (up to a 10-fold decrease), if donor fabrics or hands are dry. Another possible pathway is air-borne transmission, e.g. after shaking sheets or linen when changing bedding [62].

Although no intervention studies were identified, the IFH review includes around 19 epidemiological studies for which transmission via clothing and linens was identified as a likely cause or a significant risk factor. These included gastrointestinal and respiratory tract, together with skin and wound infections associated with clothing, shared towels (e.g., CA-MRSA), bed linen (Acinetobacter), feather pillows, and babies’ vests [63], [64], [65].

Monitoring effectiveness of laundering is another key issue. During the laundering process, temperature, duration of the wash cycle, mechanical action of water, and detergent all work together to reduce contamination levels on fabrics. In addition to physical removal, microorganisms can be killed not only by heat but also by chemical action. Other contributing factors are drying and ironing. Maintenance and care of the washer in order to prevent biofilm formation is also essential. A 2013 review by IFH comprising 29 publications on the effectiveness of laundering showed that a decrease in laundering temperature can significantly increase numbers of survivors on contaminated fabrics [66]. In situations where there were significant survivors, microbes were transferred to other items included in the wash. By contrast, efficacy can be increased, if components which release active oxygen bleach are included in the detergent formulation [67], [68], [69], [70].

A 2011 study by Lakdawalla [71] showed that up to 10^1^–10^3^ cfu/100 cm^2^ of *Clostridium difficile *could be detected on naturally contaminated bed linen even after a commercial washing process at 71°C, 3 minutes, with subsequent steam ironing in accordance with the HSG(95)18 requirements for hospital laundry arrangements for used and infected linen, U.K. Department of Health. Whether these low numbers of spores represent an infection risk is disputable, but the results suggest that there is a potential for cross-contamination of laundry during the laundry process.

A serious concern is the fact that low temperature or cold water laundering is increasingly being used in domestic settings in order to conserve energy and because many fabrics are not compatible with higher temperature laundering. Other studies are also showing that domestic washing machines often fail to reach the prescribed temperatures [72]. In private households, visibly clean laundry is perceived as being hygienically safe and falsely considered as evidence of an effective washing process. 

A major difficulty of interpreting the data in the IFH 2013 report is the extent of the variability in the results obtained from different studies under any given set of conditions. This is reflected by the diversity of recommendations for hygienic laundering of clothing in healthcare and domestic situations given by different agencies. There is an urgent need to study the impact of the key variables under carefully controlled conditions. 

A recent study shows the importance of environmental monitoring of potential infection reservoirs, and how delay in identifying a potential source of infection may increase the risk of infection. In 2013, Exner et al. (personal communication) evaluated reports of increased carriage of *K. oxyctoca* in the pediatric unit of a German hospital. An investigation revealed the existence of *K. oxytoca* in ward sinks, but hygiene interventions did not terminate the “outbreak”. It was not until further investigation when the presence of *K. oxytoca* was detected in the door seals of the washing machine, which was situated in another part of the hospital, that the probable source was identified. Retrospective study showed that only infants whose clothes were laundered in this specific machine became colonised. Following this finding, transmission could be completely stopped and the outbreak was brought under control. 

## Implications for prevention strategies

Although it is unrealistic to expect that all outbreaks of infection can be prevented, the goal is to minimise the number of outbreaks and other infection incidences, and to terminate the outbreaks which do occur as rapidly as possible. This is in accordance with the “Targeting Zero Healthcare-Associated Infections” Strategy [73], [74]. The key is the *attitude* and *commitment*, firstly to move towards zero healthcare-associated infections (HAI) despite the fact that the zero infection target will not be reached, and secondly, where infection occurs, to elucidate why it occurred. The noted U.S. infection preventionist William Jarvis argued: “Will we reach zero? No, but the attitude that I think we are moving toward, is one where clinicians don’t see these infections as inevitable. There are very sick patients who need a lot of invasive devices and procedures, so they are going to get infections. We need the attitude of trying to prevent all infections, and if one occurs, investigating to see what went wrong.” [75].

Although outbreaks are not the only concern in the prevention and control of infections, they do represent a major health hazard in hospital, long-term care facilities, and community setting. Therefore, they must be the subject of diligent investigation in all settings. Difficulties encountered in outbreak management include unpreparedness, delayed, incomplete and inconsistent analysis of infection reservoirs and routes of transmission, delayed implementation of control measures, continued spread of pathogens to other healthcare institutions, negative press and reputation, dismissal of personnel, distrust, penalties and political involvement. Successful outbreak management requires proactive precautions and reactive measures (as described, e.g., in the 2002 KRINKO recommendation [76], Table 2 (Tab. 2)). In addition, crisis management training and media training on a regular basis should be mandatory for infection control personnel. Independent regional (state) institutions responsible for adequate training of infection control personnel as “preventionists” and for coordinating and advising on outbreak management issues could be an important pillar for medical and nursing facilities as well as public facilities.

Outbreak or sporadic case reports are often published in a rush or not at all which means that valuable information cannot be retrieved and gets lost. Often, lawsuits and unfair media coverage for the institution involved are feared. Thus, apart from mandatory reports within the quality assurance system of a healthcare institution, internal reports (anonymous critical incidence report system/database) to an independent body of experts could be a way to systematically evaluate and publish important data which are then made widely accessible to all those concerned.

Targeting zero infection rates means partnering with all those affected by healthcare-associated infections, including patients and their families, hospital administrators, lawmakers, industry, and researchers. Therefore, although the definition of core competencies, legal stipulations, training and education curricula, as well as motivation of healthcare personnel are vital for the success of any infection control measures, empowerment of the patients and well-designed patient involvement programs in order to support compliance with hygiene precautions should also be promoted. This especially applies to home care settings. Educational strategies should consider “intrinsic” teaching methods apart from formal training and “extrinsic” teaching. These have been proven particularly successful with children and youth, but can be applied to adults as well. Once adequate basic hygiene techniques have become a routine, they will be kept for a lifetime. Thus, proper hygiene education during childhood is a mainstay in infection prevention, and hygiene programs for children and families should be strengthened.

## Conclusion

Future prevention strategies need to pay closer attention to the thorough investigation of infection reservoirs and routes of transmission not only from the hands, but from other sources as well. Quantitative and qualitative pathogen analysis may need to be adapted to the special challenges posed by microorganisms, which are, e.g., concealed in biofilms or entering the VBNC state. In situations where the extent of the infection risk remains uncertain, it must always be borne in mind that potential and seemingly “harmless” microbial reservoirs of pathogens (e.g., in laundry) may become an important contributing factor to severe infections or the spread of microorganisms to different settings. This is particularly the case where antibiotic-resistant strains occur or disseminate, where immunocompromised patients are involved, or where transmission pathways and low infective doses cause colonization and infection of a large number of people. Information about the detection and eradication of infection reservoirs must be made available and used to target prevention efforts, e.g., in the design of plumbing systems, water outlets, sinks and sink drains, washing machines, and novel endoscopes. The information should also be used for validating and adjusting existing infection control practices, for educating medical staff as well as patients and the general public, and for drafting new infection control protocols for previously unrecognized infection hazards. 

## Notes

### Meeting presentation 

This paper is based on the proceedings of the Rudolf Schülke Symposium held in Hamburg, 28 and 29 November 2013.

### Competing interests

The authors declare that they have no competing interests.

## Figures and Tables

**Table 1:**
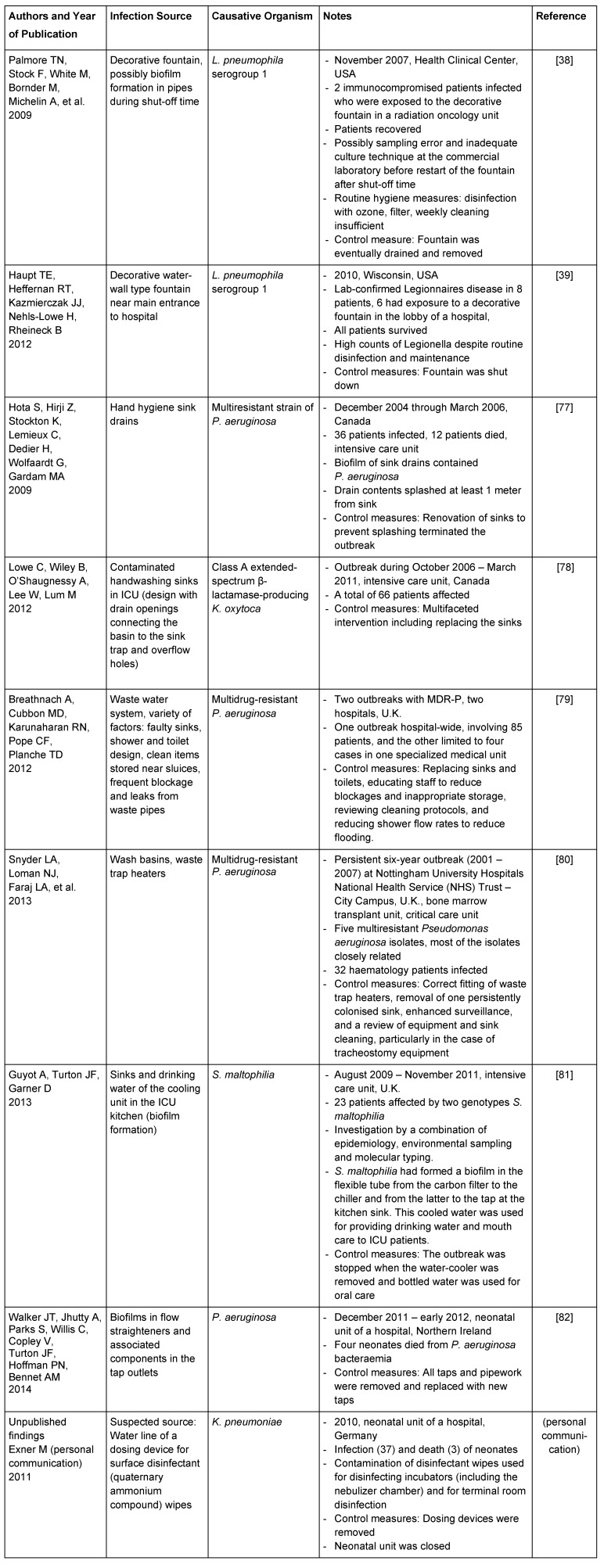
Examples for water-associated infection reservoirs in healthcare institutions

**Table 2:**
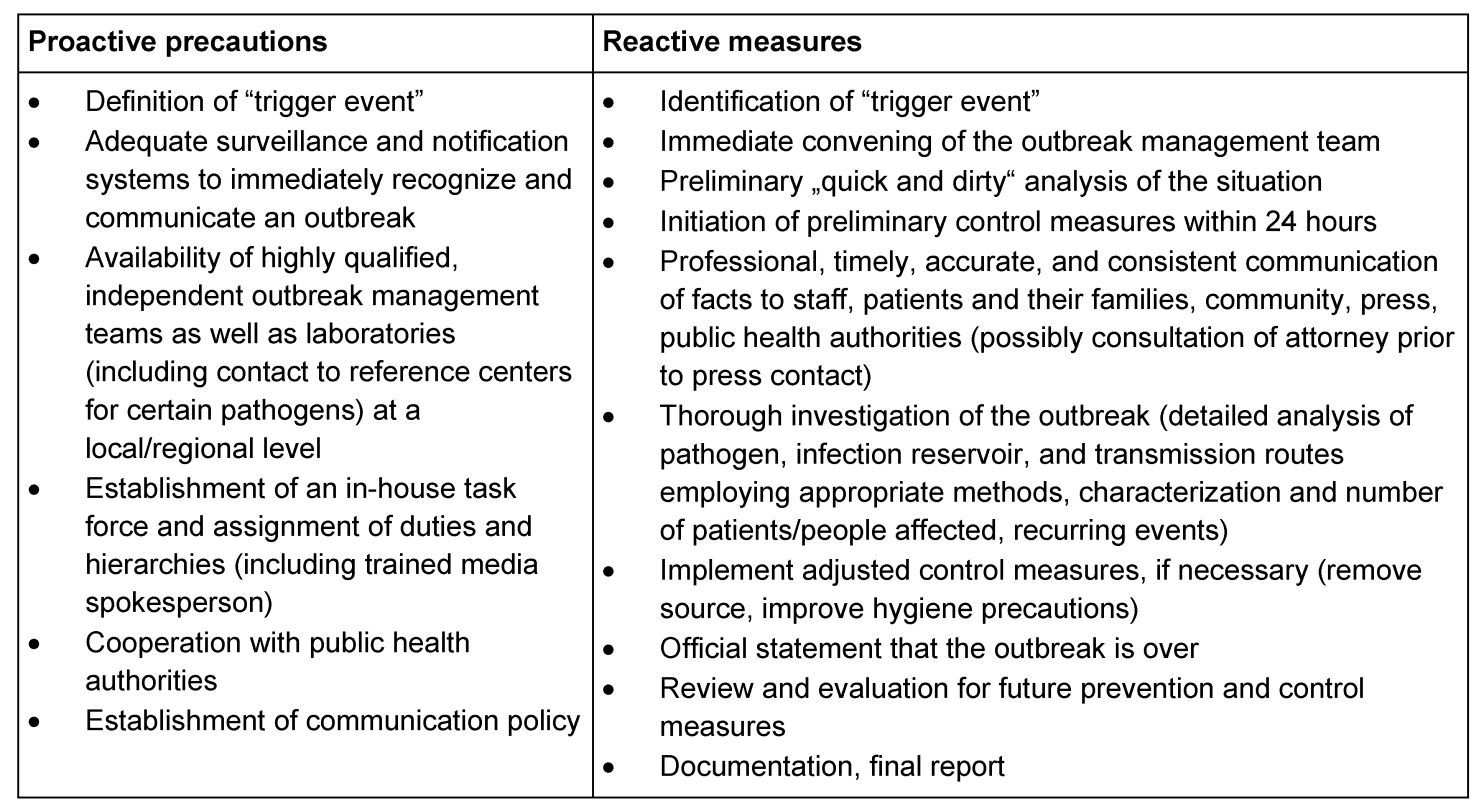
Proactive and reactive measures in outbreak management (based on KRINKO [76])
